# Dynamics of a discrete-time predator-prey model with exponential prey growth and saturated response

**DOI:** 10.1038/s41598-026-41693-y

**Published:** 2026-03-21

**Authors:** Hala H. Emam, H. El-Metwally, M. Y. Hamada

**Affiliations:** 1 Department of Mathematics, Faculty of Science, Mansoura University, Mansoura, Egypt; 2 Mathematics Department, Faculty of Engineering, German International University, Cairo, Egypt; 3 Department of Basic Science, High Institute for Engineering and Technology, Al-Obour, Egypt

**Keywords:** Discrete predator-prey, Equilibrium points, Period-doubling bifurcation, Neimark–Sacker bifurcation, Chaos, Ecology, Ecology, Mathematics and computing, Physics

## Abstract

Recent studies have increasingly focused on the stability of predator-prey systems incorporating the Holling functional response and Ricker population model. This work investigates the influence of the Holling effect on a discrete-time predator-prey model, demonstrating through bifurcation theory and the central manifold theorem that the system exhibits period-doubling and Neimark-Sacker bifurcations at equilibrium points. Numerical simulations reveal complex dynamical behaviors, with bifurcation diagrams illustrating transitions from stability to periodic oscillations and chaos. Using phase portraits, Lyapunov exponents, and bifurcation analysis, we show how the Ricker map progresses from order to chaos. Our findings enhance the understanding of predator-prey dynamics and provide insights for ecological population management, highlighting the system’s rich behavior under parameter variations.

## Introduction

In ecological systems, species are organized into distinct trophic levels, defined by their feeding relationships and physiological characteristics. These species interact in various complex ways, and among these interactions, the predator-prey relationship is one of the most fundamental and widely studied in population dynamics. Predators influence not only the abundance of prey but also the fluctuations within their own populations. These reciprocal effects are key to understanding population regulation and stability within ecosystems. Due to the complexity of such interactions, mathematical modeling has become an essential tool for their analysis^1^.

Predator-prey models have long been central to the study of mathematical biology, offering a structured approach to understanding biological interactions. These models commonly explore factors such as population size, age distribution, and environmental influences. Kuang and Beretta^2^ showed that ratio-dependent models exhibit more intricate boundary dynamics than classical predator-prey systems, and avoid certain paradoxes such as enrichment and biological control. Subsequent research has focused on models demonstrating periodic or chaotic behavior^3,4^. These insights allow researchers to predict how variations in prey or predator populations might influence the broader ecosystem. For example, predator-prey models are valuable in fisheries management, where they help assess the ecosystem-wide consequences of harvesting beyond the targeted species^5^.

Population dynamics are commonly studied through either continuous-time or discrete-time models. While many classical models adopt a continuous-time framework^6,7^, discrete-time models have also proven to be powerful, especially in cases involving species with non-overlapping generations^8–12^. Freedman^13^ emphasized that discrete-time models are more appropriate when population events, such as births and deaths, occur at distinct intervals. In addition to being more suitable for certain ecological scenarios, discrete-time models often exhibit richer dynamical behavior and are more amenable to numerical analysis.

An essential component of predator-prey modeling is the functional response, which characterizes the relationship between a predator’s consumption rate and prey density. Among the various types, the Holling type II functional response is one of the most widely used. It describes a scenario where the predator’s feeding rate increases with prey density but eventually saturates due to handling time constraints. This functional response has been shown to effectively describe the behavior of many predators, including birds, mammals, and insects^5^. Depending on parameter values, it can either stabilize or destabilize the predator-prey system.

The integration of the Ricker model with the Holling type II functional response provides a robust framework for analyzing the dynamics of predator-prey interactions under various ecological and management scenarios^14^. Moreover, discrete-time systems with such structures are known to exhibit complex bifurcation phenomena, including flip and Neimark–Sacker bifurcations^15^, which are critical in understanding the transition to chaos or stable oscillations.

There is a significant difference in the dynamics of the Ricker model $rx_{n}e^{1-x_{n}}$ that is utilized in predator-prey models. The variable *x* in the Ricker model, which denotes the number of prey, can assume any real number that is positive. Because of this flexibility, there is no need to set restrictions on the parameters in order to guarantee this variability.

Our model’s dynamical range is increased by using the Ricker map, which allows us to capture oscillations that more closely resemble actual variations in prey and predator populations. This addition improves our model’s capacity to replicate the intricacies of natural ecosystems, offering a more sophisticated comprehension of population dynamics and predator-prey relationships.

Volterra developed a system of two ordinary differential equations^16^ to represent the relationship between predators and their prey in an ecological system. The Volterra system is the most basic model of predator-prey relationships, based on linear per capita growth rates. This system is an example of the well-known Volterra predator-prey model.

$$\begin{aligned} \dot{X}= & aX-bXY \\ \dot{Y}= & -cY+dXY, \end{aligned}$$

where *Y* represents the number of predators and *X* represents the number of prey. The parameters *a*, *b*, *c*, and *d* are also all positive. It has been shown that the number of prey grows exponentially when there are no predators around, whereas the number of predators decreases exponentially when there are no prey. Predator-prey interactions that are beneficial to predators but fatal to prey are referred to as *bXY* and *dXY*. This model would be well updated by substituting the logistic growth $ax(1-x)$ for the term *aX*, where the population’s per capita growth rate decreases as the population size gets closer to a limit imposed by limited resources. Nonetheless, we are quite interested in examining the impact of describing prey dynamics using the Ricker map rather than the logistic map. The population growth in the Ricker map is almost exponential, but as the population rises, the rapid growth rate falls linearly. Eventually, the population size reaches an average and oscillates around a mean. This model has been successfully applied to describe the population dynamics of a variety of species, including mammals, and has been extended to incorporate different functional responses^17–19^.

After set of Developments to Volterra model, the following discrete-time prey-predator population model^20^

0.1 $$\begin{aligned} x_{n+1}= & rx_{n}e^{1-x_{n}}-\frac{\alpha x_{n}y_{n}}{1+x_{n}}, \nonumber \\ y_{n+1}= & \frac{\alpha x_{n}y_{n}}{1+x_{n}}, \end{aligned}$$

where *r* and $\alpha$ are positive parameters, $rx_{n}e^{1-x_{n}}$ refers to the logistic growth of prey, when the predator absent, and the term $\frac{\alpha x_{n}y_{n}}{1+x_{n}}$ is a Holling type $\amalg$ function, which describes the decrease of prey due to predation.

This study focuses on a discrete-time predator-prey model that combines the Ricker growth mechanism for the prey population with the Holling type II functional response for the predator. The Ricker model is selected for its simplicity and ability to capture essential features of density-dependent population growth. The Holling type II response is used to reflect the saturation effect in predator feeding behavior at high prey densities. By merging these two components, we aim to explore the qualitative dynamics, stability, and bifurcation behaviors of the system, contributing to both theoretical ecology and practical conservation modeling.

A discrete-time predator-prey model is described by the following system of difference equations:

0.2 $$\begin{aligned} \begin{aligned} x_{n+1}&= x_{n} + r x_{n} e^{1 - x_{n}} - \frac{\alpha x_{n} y_{n}}{1 + x_{n}}, \\ y_{n+1}&= y_{n} + \frac{\alpha x_{n} y_{n}}{1 + x_{n}} - \gamma y_{n}. \end{aligned} \end{aligned}$$

In model (0.2), the parameters *r*, $\alpha$, and $\gamma$ are positive constants, and each term represents a specific ecological process:

The term $r x_{n} e^{1 - x_{n}}$ describes the prey’s population growth in the absence of predation, following a Ricker-type exponential growth. - The term $\frac{\alpha x_{n} y_{n}}{1 + x_{n}}$ represents the reduction in prey due to predation, modeled using a Holling type II functional response. This expression also contributes to the predator’s growth, as it captures the rate at which predators consume prey. - The term $\gamma y_{n}$ accounts for the natural mortality of the predator population.

This paper is organized as follows: In Section "Existence and stability of fixed points", we analyze the existence and local stability of the equilibrium points of model(0.2). Section “Bifurcation analysis” investigates the occurrence of flip bifurcation and Neimark–Sacker bifurcation using $r^*$ as the bifurcation parameter. In Section “Numerical study”, numerical simulations are presented to support and illustrate the theoretical findings. The paper concludes with a summary of the main results in the final section.

## Existence and stability of fixed points

In this section, we analyze the existence and local stability of the fixed points of the discrete dynamical system described by Equation (0.2). The fixed points of the map (0.2) are characterized as follows:

### Proposition 1.1

For the system defined by Equation (0.2), depending on the parameters *r*, $\alpha$, and $\gamma$, there exist two possible fixed points:

1. The trivial fixed point $p_0 = (0,0)$ always exists.
2. A non-trivial (interior) fixed point
  $$p_1 = \left( \frac{\gamma }{\alpha - \gamma }, \frac{r}{\alpha - \gamma } e^{\frac{\alpha - 2\gamma }{\alpha - \gamma }} \right)$$
  exists if and only if $\alpha> \gamma$.

### Proof

Fixed points correspond to steady states of the map (0.2), which are solutions to the system:

$$\begin{aligned} x&= x + rxe^{1-x} - \frac{\alpha xy}{1+x}, \\ y&= y + \frac{\alpha xy}{1+x} - \gamma y. \end{aligned}$$

Solving the above system yields two non-negative fixed points: the trivial point $p_0 = (0,0)$, and the interior point

$$p_1 = \left( \frac{\gamma }{\alpha - \gamma }, \frac{r}{\alpha - \gamma } e^{\frac{\alpha - 2\gamma }{\alpha - \gamma }} \right) ,$$

which exists if and only if $\alpha> \gamma$. $\square$

Biologically, $p_0$ corresponds to extinction (absence of both species), while $p_1$ represents a coexistence state with positive predator and prey densities.

To assess the local stability of these fixed points, we linearize the system by computing the Jacobian matrix *J*(*x*, *y*) at an arbitrary point (*x*, *y*):

1.1 $$\begin{aligned} J(x, y) = \begin{pmatrix} \displaystyle 1 + re^{1-x}(1 - x) - \frac{\alpha y}{(1+x)^2} & -\frac{\alpha x}{1+x} \\ \frac{\alpha y}{(1+x)^2} & (1 - \gamma ) + \frac{\alpha x}{1+x} \end{pmatrix}. \end{aligned}$$

The corresponding characteristic polynomial is:

1.2 $$\begin{aligned} R(\rho ) = \rho ^2 - P(x, y)\rho + q(x, y), \end{aligned}$$

where

$$\begin{aligned} P(x, y)&= \text {tr}(J(x, y)) = 2 - \gamma + re^{1-x}(1 - x) + \frac{\alpha x}{1 + x} - \frac{\alpha y}{(1 + x)^2}, \\ q(x, y)&= \det (J(x, y)) = \left( 1 - \gamma + \frac{\alpha x}{1 + x} \right) \left( 1 + re^{1-x}(1 - x) - \frac{\alpha y}{(1 + x)^2} \right) + \frac{\alpha ^2 x y}{(1 + x)^3}. \end{aligned}$$

To interpret the eigenvalues $\rho _1$ and $\rho _2$ of (1.2), we use the following classification:

### Definition 1.1

Let $p = (x, y)$ be a fixed point of the system (0.2), and let $\rho _1$, $\rho _2$ be the eigenvalues of the Jacobian matrix at *p*.

1. *p* is *locally asymptotically stable (LAS)* if $|\rho _1| < 1$ and $|\rho _2| < 1$.
2. *p* is a *source* if $|\rho _1|> 1$ and $|\rho _2|> 1$.
3. *p* is a *saddle point* (SP) if exactly one of $|\rho _1|$, $|\rho _2|$ is greater than 1.
4. *p* is *non-hyperbolic (NHP)* if $|\rho _1| = 1$ or $|\rho _2| = 1$.

The following lemma gives a test for stability based on the coefficients of the characteristic polynomial (1.2).

### Lemma 1.1

Let $R(\rho ) = \rho ^2 + A\rho + B$, and suppose $R(1)> 0$. Then the following statements hold:

1. $|\rho _1| < 1$, $|\rho _2| < 1$ if and only if $R(-1)> 0$ and $B < 1$;
2. $|\rho _1|< 1 < |\rho _2|$ (or vice versa) if and only if $R(-1) < 0$;
3. $|\rho _1|> 1$, $|\rho _2|> 1$ if and only if $R(-1)> 0$ and $B> 1$;
4. $\rho _1 = -1$, $|\rho _2| \ne 1$ if and only if $R(-1) = 0$ and $B \ne 0, 2$;
5. $\rho _1$ and $\rho _2$ are complex conjugates with $|\rho _1| = |\rho _2| = 1$ if and only if $A^2 - 4B < 0$ and $B = 1$.

### Stability of the trivial fixed point $p_0$

#### Proposition 1.2

The stability of the trivial fixed point $p_0 = (0,0)$ in system (0.2) is characterized as follows:

1. $p_0$ constitutes a stable fixed point if and only if $\frac{1}{r}>\frac{e\left( 1-\gamma \right) }{\gamma }$;
2. $p_0$ is an unstable fixed point if and only if $\frac{1}{r}<\frac{e\left( 1-\gamma \right) }{\gamma }$;
3. $p_0$ is never a saddle and non-hyperbolic.

#### Proof

At $p_0 = (0,0)$, the Jacobian matrix simplifies, and the characteristic polynomial becomes:

$$R(\rho ) = \rho ^2 - P(0,0)\rho + q(0,0),$$

where

$$P(0,0) = 2 - \gamma + re, \quad q(0,0) = (1 - \gamma )(1 + re).$$

Using Lemma 1.1, $p_0$ is stable (a sink) if:

$$\begin{aligned} R(-1)&= 1 + P(0,0) + q(0,0)> 0, \\ q(0,0)&< 1. \end{aligned}$$

Substituting in the expressions yields the condition:

$$re(1 - \gamma ) < \gamma .$$

The point is unstable (a source) if $re(1 - \gamma )> \gamma$. Saddle or non-hyperbolic behavior is excluded as this contradict with *r* is always positive value. $\square$

### Stability of the interior fixed point $p_1$

#### Proposition 1.3

The interior fixed point $p_1$ is locally asymptotically stable if and only if the following conditions are satisfied:

1. $$re^{\frac{\alpha - 2\gamma }{\alpha - \gamma }} \cdot \left( \frac{\gamma \left( \gamma ^{2}+\alpha ^{2}-2\alpha \gamma \right) }{\alpha \left( \alpha -\gamma \right) }\right)> 0$$;
2. $$4 + re^{\frac{\alpha - 2\gamma }{\alpha - \gamma }} \cdot \left( \frac{\gamma \left( \gamma ^{2}-2\gamma +\alpha ^{2}-2\alpha \gamma \right) }{\alpha \left( \alpha -\gamma \right) }\right)> 0$$;
3. $$re^{\frac{\alpha - 2\gamma }{\alpha - \gamma }} \cdot \left( \frac{\gamma \left( \gamma ^{2}-\gamma +\alpha ^{2}-2\alpha \gamma \right) }{\alpha \left( \alpha -\gamma \right) }\right) < 0$$.

#### Proof

The Jacobian matrix evaluated at $p_1$ yields a characteristic polynomial:

$$\rho ^2 + P \rho + q = 0,$$

with

$$P = \left( \frac{\gamma ^2}{\alpha ^2 - \alpha \gamma } \right) re^{\frac{\alpha - 2\gamma }{\alpha - \gamma }} - 2,$$

and

$$q = \left( \frac{\gamma ^3 - \gamma ^2 + \gamma \alpha ^2 - 2\alpha \gamma ^2}{\alpha ^2 - \alpha \gamma } \right) re^{\frac{\alpha - 2\gamma }{\alpha - \gamma }} + 1.$$

Using standard results on second-order stability, the point $p_1$ is stable if:

$$|P|< 1 + q < 2.$$

This inequality gives rise to the three conditions listed below

1. $$1+P+q>0,$$ we get $$re^{\frac{\alpha -2\gamma }{\alpha -\gamma }}\left( \frac{\gamma ^{3}+\gamma \alpha ^{2}-2\alpha \gamma ^{2}}{ \alpha ^{2}-\alpha \gamma }\right)>0;$$
2. $1-P+q>0$, this leads to $4+re^{\frac{\alpha -2\gamma }{ \alpha -\gamma }}\left( \frac{\gamma ^{3}-2\gamma ^{2}+\gamma \alpha ^{2}-2\alpha \gamma ^{2}}{\alpha ^{2}-\alpha \gamma }\right)>0;$
3. $$q<1$$, translates as $$re^{\frac{\alpha -2\gamma }{\alpha -\gamma }}\left( \frac{\gamma ^{3}-\gamma ^{2}+\gamma \alpha ^{2}-2\alpha \gamma ^{2}}{\alpha ^{2}-\alpha \gamma }\right) <0.$$

$$\square $$

### Loss of stability of the interior fixed point

#### Proposition 1.4

The interior fixed point $p_1$ loses stability through:

1. **Period-doubling bifurcation** if:
  - $$re^{\frac{\alpha - 2\gamma }{\alpha - \gamma }} \cdot \left( \frac{\gamma \left( \gamma ^{2}+\alpha ^{2}-2\alpha \gamma \right) }{\alpha \left( \alpha -\gamma \right) }\right) = 0$$;
  - $$\left( \frac{\gamma \left( \gamma ^{2}-\gamma +\alpha ^{2}-2\alpha \gamma \right) }{\alpha \left( \alpha -\gamma \right) }\right) re^{\frac{ \alpha -2\gamma }{\alpha -\gamma }}+1>0;$$.
2. **Neimark–Sacker bifurcation** if:
  - $$\left| \left( \frac{\gamma ^{2}}{\alpha ^{2}-\alpha \gamma }\right) re^{\frac{\alpha -2\gamma }{\alpha -\gamma }}-2\right| -1<\left( \frac{\gamma \left( \gamma ^{2}-\gamma +\alpha ^{2}-2\alpha \gamma \right) }{\alpha \left( \alpha -\gamma \right) }\right) re^{\frac{\alpha -2\gamma }{\alpha -\gamma }}+1;$$
  - $$\left( \frac{\gamma \left( \gamma ^{2}-\gamma +\alpha ^{2}-2\alpha \gamma \right) }{\alpha \left( \alpha -\gamma \right) }\right) re^{\frac{ \alpha -2\gamma }{\alpha -\gamma }}=0.$$

#### Proof

According to standard bifurcation theory^21^, a period-doubling occurs when $1 + P + q = 0$ and $q> 0$. Solving $1 + P + q = 0$ under the expression of *P* and *q* yields $re^{\frac{\alpha -2\gamma }{\alpha -\gamma }}\left( \frac{\gamma ^{3}+\gamma \alpha ^{2}-2\alpha \gamma ^{2}}{ \alpha ^{2}-\alpha \gamma }\right) =0$ and $q = \left( \frac{\gamma ^3 - \gamma ^2 + \gamma \alpha ^2 - 2\alpha \gamma ^2}{\alpha ^2 - \alpha \gamma } \right) re^{\frac{\alpha - 2\gamma }{\alpha - \gamma }} + 1>0.$

The Neimark–Sacker (torus) bifurcation occurs when the eigenvalues become complex conjugates with modulus 1, which corresponds to $q = 1$ and the magnitude condition on *P*, $\left( i.e\right)$ $\left( \left| p\right| -1<q\right)$ and $q=1$. $\square$

## Bifurcation analysis

In this section, we analyze the occurrence of bifurcations near the fixed point $p_1$ of model (0.2), using the theoretical results established in Section “Introduction”. The classification of bifurcation types is determined primarily by the nature of the eigenvalues of the Jacobian matrix evaluated at the fixed points.

### Neimark–Sacker bifurcation at $p_1$

We investigate the Neimark–Sacker bifurcation of model (0.2) at the fixed point $p_1$. To this end, we consider the parameter *r* varying in a small neighborhood of a critical value $r^{*}$, and write

$$r = r^{*} + \epsilon , \quad \text {with } \epsilon \ll 1.$$

Substituting this into model (0.2), we obtain:

2.1 $$\begin{aligned} x_{n+1}&= x_n + (r^{*} + \epsilon )x_n e^{1 - x_n} - \frac{\alpha x_n y_n}{1 + x_n}, \nonumber \\ y_{n+1}&= y_n + \frac{\alpha x_n y_n}{1 + x_n} - \gamma y_n. \end{aligned}$$

Let us compute the Jacobian matrix *J*(*x*, *y*) evaluated at the fixed point

$$p_1 = \left( \frac{\gamma }{\alpha - \gamma }, \frac{r}{\alpha - \gamma } e^{\frac{\alpha - 2\gamma }{\alpha - \gamma }} \right) .$$

The characteristic equation of the Jacobian matrix is:

$$\rho ^2 - P(\epsilon )\rho + q(\epsilon ) = 0,$$

where

$$P(\epsilon ) = 2 - (r^{*} + \epsilon ) \left( \frac{\gamma ^2}{\alpha ^2 - \alpha \gamma } \right) e^{\frac{\alpha - 2\gamma }{\alpha - \gamma }},$$

and

$$q(\epsilon ) = \left( \frac{\gamma ^3 - \gamma ^2 + \gamma \alpha ^2 - 2\alpha \gamma ^2}{\alpha ^2 - \alpha \gamma } \right) (r^{*} + \epsilon ) e^{\frac{\alpha - 2\gamma }{\alpha - \gamma }} + 1.$$

The eigenvalues of the Jacobian at $p_1$ are given by:

$$\begin{aligned} \rho _{1,2}&= \frac{P(\epsilon ) \pm i\sqrt{4q(\epsilon ) - P^2(\epsilon )}}{2} \\&= \left( 1 - (r^{*} + \epsilon ) \left( \frac{\gamma ^2}{2\alpha (\alpha - \gamma )} \right) e^{\frac{\alpha - 2\gamma }{\alpha - \gamma }} \right) \quad \pm \frac{i}{2} \sqrt{ \frac{(r^{*} + \epsilon )e^{\frac{\alpha - 2\gamma }{\alpha - \gamma }} \left[ 4\alpha (\alpha - \gamma )(\gamma ^3 + \gamma \alpha ^2 - 2\alpha \gamma ^2) - \gamma ^4 (r^{*} + \epsilon ) e^{\frac{\alpha - 2\gamma }{\alpha - \gamma }} \right] }{ \alpha ^2 (\alpha - \gamma )^2 } }. \end{aligned}$$

The modulus of these eigenvalues is:

$$|\rho _{1,2}| = \sqrt{q(\epsilon )} = \sqrt{ \left( \frac{\gamma ^3 - \gamma ^2 + \gamma \alpha ^2 - 2\alpha \gamma ^2}{\alpha (\alpha - \gamma )} \right) (r^{*} + \epsilon ) e^{\frac{\alpha - 2\gamma }{\alpha - \gamma }} + 1 }.$$

Differentiating with respect to $\epsilon$ and evaluating at $\epsilon = 0$ gives:

$$\frac{d\left| \rho _{1,2}\right| }{d\varepsilon }=e^{\frac{\alpha -2\gamma }{2\alpha -2\gamma }}\sqrt{\frac{\left( \gamma ^{3}-\gamma ^{2}+\gamma \alpha ^{2}-3\alpha \gamma ^{2}\right) ^{2}}{4\alpha ^{2}\left( \alpha -\gamma \right) ^{2}e^{\frac{2\gamma -\alpha }{2\alpha -2\gamma } }+4r^{*}\alpha \left( \alpha -\gamma \right) \left( \gamma ^{3}-\gamma ^{2}+\gamma \alpha ^{2}-2\alpha \gamma ^{2}\right) }}>0.$$

$$\left. \frac{d|\rho _{1,2}|}{d\epsilon } \right| _{\epsilon =0}> 0.$$

This ensures that the modulus of the eigenvalues crosses the unit circle transversally.

Additionally, by evaluating the characteristic polynomial at $\epsilon = 0$, we find that $\rho _{1,2}^m \ne 1$ for $m = 1, 2, 3, 4$, implying that $P(0) \notin \{-2, 0, 1, 2\}$, which rules out strong resonances.

To study the local dynamics near $p_1$, we define new variables:

$$u_n = x_n - x^{*}, \quad v_n = y_n - y^{*},$$

where $$x^{*} = \frac{\gamma }{\alpha - \gamma }$$ and $$y^{*} = \frac{r}{\alpha - \gamma } e^{\frac{\alpha - 2\gamma }{\alpha - \gamma }}$$.

Model (2.1) then transforms into:

2.2 $$\begin{aligned} u_{n+1}&= u_n + (r^{*} + \epsilon )(u_n + x^{*}) e^{1 - u_n - x^{*}} - \frac{\alpha (u_n + x^{*})(v_n + y^{*})}{1 + u_n + x^{*}}, \nonumber \\ v_{n+1}&= v_n + \frac{\alpha (u_n + x^{*})(v_n + y^{*})}{1 + u_n + x^{*}} - \gamma (v_n + y^{*}). \end{aligned}$$

We expand system (2.2) in a Taylor series around $(u_n, v_n) = (0, 0)$ up to third order:

$$\begin{aligned} u_{n+1}&= B_{11} u_n + B_{12} v_n + B_{13} u_n^2 + B_{14} u_n v_n + B_{15} u_n^3 + B_{16} u_n^2 v_n + \mathcal {O}(\Vert (u_n, v_n)\Vert ^4), \\ v_{n+1}&= B_{21} u_n + B_{22} v_n + B_{23} u_n^2 + B_{24} u_n v_n + B_{25} u_n^3 + B_{26} u_n^2 v_n + \mathcal {O}(\Vert (u_n, v_n)\Vert ^4), \end{aligned}$$

where the coefficients $B_{ij}$ are explicitly defined in terms of $x^{*}$, $y^{*}$, $r^{*}$, and the model parameters $\alpha$, $\gamma$.

$$B_{11}=\frac{-r^{*}\left( x^{*}-1\right) \left( x^{*}+1\right) ^{2}e^{1-x^{*}}-\alpha y^{*}}{\left( x^{*}+1\right) ^{2}}+2,$$ $$\ \ \ \ \ \ \ \ \ \ \ \quad B_{12}=\frac{-\alpha x^{*}}{x^{*}+1},$$

$$B_{13}=\frac{\left( x^{*}+1\right) ^{3}\left( x^{*}-2\right) r^{*}e^{1-x^{*}}+2\alpha y^{*}}{2\left( x^{*}+1\right) ^{3}} ,$$ $$B_{14}=\frac{-\alpha }{\left( x^{*}+1\right) ^{2}},$$

$$B_{15}=\frac{-\left( x^{*}+1\right) ^{4}\left( x^{*}-3\right) r^{*}e^{1-x^{*}}-6\alpha y^{*}}{6\left( x^{*}+1\right) ^{4}},$$ $$B_{16}=\frac{\alpha }{\left( x^{*}+1\right) ^{3}},$$

$$B_{21}=\frac{\alpha y^{*}}{\left( x^{*}+1\right) ^{2}},$$ $$B_{22}= \frac{\alpha x^{*}}{x^{*}+1}+1,$$

$$B_{23}=\frac{-\alpha y^{*}}{\left( x^{*}+1\right) ^{3}},$$ $$B_{24}= \frac{\alpha }{\left( x^{*}+1\right) ^{2}},$$

$$B_{25}=\frac{\alpha y^{*}}{\left( x^{*}+1\right) ^{4}},$$ $$B_{26}= \frac{-\alpha }{\left( x^{*}+1\right) ^{3}}.$$

To transform this system into its normal form, we define the variables $(X_n, Y_n)$ by the linear transformation:

$$\begin{pmatrix} u_n \\ v_n \end{pmatrix} = \begin{pmatrix} B_{12} & 0 \\ \eta - B_{11} & -\zeta \end{pmatrix} \begin{pmatrix} X_n \\ Y_n \end{pmatrix},$$

where

$$\eta = 1 - \left( \frac{\gamma ^2}{2\alpha (\alpha - \gamma )} \right) (r^{*} + \epsilon ) e^{\frac{\alpha - 2\gamma }{\alpha - \gamma }}, \quad \zeta = \frac{1}{2} \sqrt{ \frac{ (r^{*} + \epsilon ) e^{\frac{\alpha - 2\gamma }{\alpha - \gamma }} [4\alpha (\alpha - \gamma )(\gamma ^3 + \gamma \alpha ^2 - 2\alpha \gamma ^2) - \gamma ^4 (r^{*} + \epsilon ) e^{\frac{\alpha - 2\gamma }{\alpha - \gamma }} ] }{ \alpha ^2 (\alpha - \gamma )^2 } }.$$

Then system (2.2) becomes:

2.3 $$\begin{aligned} \begin{pmatrix} X_{n+1} \\ Y_{n+1} \end{pmatrix} = \begin{pmatrix} \eta & -\zeta \\ \zeta & \eta \end{pmatrix} \begin{pmatrix} X_n \\ Y_n \end{pmatrix} + \begin{pmatrix} \Psi (X_n, Y_n) \\ \Phi (X_n, Y_n) \end{pmatrix}, \end{aligned}$$

where $\Psi$ and $\Phi$ are polynomials containing the nonlinear terms of order $\ge 2$.

2.4 $$\begin{aligned} \Psi \left( X_{n},Y_{n}\right)= & A_{11}X_{n}^{3}+A_{12}X_{n}Y_{n}+A_{13}X_{n}^{2}+A_{14}X_{n}^{2}Y_{n}+O \left( \left| X_{n}\right| ,\left| Y_{n}\right| \right) ^{4}, \nonumber \\ \Phi \left( X_{n},Y_{n}\right)= & A_{21}X_{n}^{3}+A_{22}X_{n}Y_{n}+A_{23}X_{n}^{2}+A_{24}X_{n}^{2}Y_{n}+O \left( \left| X_{n}\right| ,\left| Y_{n}\right| \right) ^{4}, \end{aligned}$$

where

$$\begin{aligned} A_{11}&= -B_{12} \bigl (B_{12} B_{15} + B_{16}(\eta - B_{11})\bigr ), \quad&A_{12}&= -\zeta B_{14}, \\ A_{13}&= B_{12} B_{13} + B_{14}(\eta - B_{11}), \quad&A_{14}&= \zeta B_{12} B_{16}, \\ A_{21}&= \frac{B_{12}\bigl (-B_{25} B_{12}^2 + B_{12}(B_{15} - B_{26})(\eta - B_{11}) + B_{16}(\eta - B_{11})^2\bigr )}{\zeta }, \quad&A_{22}&= B_{12} B_{24} + B_{14}(\eta - B_{11}), \\ A_{23}&= \frac{-B_{23} B_{12}^2 + B_{12}(B_{13} - B_{24})(\eta - B_{11}) + B_{14}(\eta - B_{11})^2}{\zeta }, \quad&A_{24}&= B_{12} \bigl (B_{12} B_{26} + B_{16}(\eta - B_{11})\bigr ). & \end{aligned}$$

$$\begin{aligned} \Psi _{X_n X_n} \big |_{(0,0)}&= 2A_{13}, \quad&\Psi _{Y_n Y_n} \big |_{(0,0)}&= 0, \quad&\Psi _{X_n Y_n} \big |_{(0,0)}&= A_{12}, \\ \Psi _{X_n X_n Y_n} \big |_{(0,0)}&= 2A_{14}, \quad&\Psi _{X_n X_n X_n} \big |_{(0,0)}&= 6A_{11}, \quad&\Psi _{Y_n Y_n Y_n} \big |_{(0,0)} = \Psi _{X_n Y_n Y_n} \big |_{(0,0)} = 0. \end{aligned}$$

$$\begin{aligned} \Phi _{X_n X_n} \big |_{(0,0)}&= 2A_{23}, \quad&\Phi _{Y_n Y_n} \big |_{(0,0)}&= 0, \quad&\Phi _{X_n Y_n} \big |_{(0,0)}&= A_{22}, \\ \Phi _{X_n X_n Y_n} \big |_{(0,0)}&= 2A_{24}, \quad&\Phi _{X_n X_n X_n} \big |_{(0,0)}&= 6A_{21}, \quad&\Phi _{Y_n Y_n Y_n} \big |_{(0,0)} = \Phi _{X_n Y_n Y_n} \big |_{(0,0)} = 0. \end{aligned}$$

The normal form analysis of (2.3) shows that a Neimark–Sacker bifurcation occurs at $p_1$ if the first Lyapunov coefficient $\chi$ is nonzero. This coefficient $\chi$ is computed explicitly in terms of the $A_{ij}$ coefficients, which are themselves functions of the $B_{ij}$. The signs of $\chi$ and $d|\rho |/d\epsilon$ determine the direction and stability of the bifurcating invariant closed curve.

$$\chi =-\text {Re}\left[ \frac{\left( 1-2\overline{\rho }\right) \overline{ \rho }^{2}}{1-\rho }\delta _{11}\delta _{20}\right] -\frac{1}{2}\left\| \delta _{11}\right\| ^{2}-\left\| \delta _{02}\right\| ^{2}+\text {Re }\left( \overline{\rho }\delta _{21}\right) ,$$

such that

$$\delta _{11}=\frac{1}{4}\left[ \Psi _{X_{n}X_{n}}+\ \Psi _{Y_{n}Y_{n}}+l\left( \Phi _{X_{n}X_{n}}+\Phi _{Y_{n}Y_{n}}\right) \right] |_{\left( 0,0\right) },$$

$$\delta _{20}=\frac{1}{8}\left[ \Psi _{X_{n}X_{n}}-\ \Psi _{Y_{n}Y_{n}}+2\ \Phi _{X_{n}Y_{n}}+l\left( \Phi _{X_{n}X_{n}}-\Phi _{Y_{n}Y_{n}}-2\Psi _{X_{n}Y_{n}}\right) \right] |_{\left( 0,0\right) },$$

$$\delta _{02}=\frac{1}{8}\left[ \Psi _{X_{n}X_{n}}-\ \Psi _{Y_{n}Y_{n}}+2\ \Phi _{X_{n}Y_{n}}+l\left( \Phi _{X_{n}X_{n}}-\Phi _{Y_{n}Y_{n}}+2\Psi _{X_{n}Y_{n}}\right) \right] |_{\left( 0,0\right) },$$

$$\delta _{21}=\frac{1}{16}\left[ \begin{array}{c} \ \Psi _{X_{n}X_{n}X_{n}}+\Psi _{X_{n}Y_{n}Y_{n}}+\Phi _{X_{n}X_{n}Y_{n}}+\Phi _{Y_{n}Y_{n}Y_{n}} \\ +l\left( \Phi _{X_{n}X_{n}X_{n}}+\Phi _{X_{n}Y_{n}Y_{n}}-\Psi _{X_{n}X_{n}Y_{n}}-\Psi _{Y_{n}Y_{n}Y_{n}}\right) \end{array} \right] |_{\left( 0,0\right) },$$

after calculating, we obtain

$$\delta _{11}=\frac{1}{2}\left( A_{13}+lA_{23}\right) ,$$

$$\delta _{20}=\frac{1}{4}\left[ A_{13}+A_{22}+l\left( A_{23}-A_{12}\right) \right] ,$$

$$\delta _{02}=\frac{1}{4}\left[ A_{13}+A_{22}+l\left( A_{23}+A_{12}\right) \right] ,$$

$$\delta _{21}=\frac{1}{8}\left[ 3A_{11}+A_{24}+l\left( 3A_{21}-A_{24}\right) \right] .$$

After understanding the system and using the Neimark-Sacker bifurcation proposition discussed in^22,23^, we can propose the following proposition:

#### Proposition 2.1

Assuming $\chi \ne 0$, the model (0.2) has a Neimark–Sacker bifurcation around $p_{1}\left( \frac{\gamma }{\alpha -\gamma },\frac{r}{ \alpha -\gamma }e^{\frac{\alpha -2\gamma }{\alpha -\gamma }}\right)$. Furthermore, if $\chi <0$ $\left( \text {resp. }\chi>0\right)$, an attracting (resp. repelling) closed curve emerges from $p_{1}.$

### Period-doubling bifurcation analysis

In this section, we analyze the period-doubling bifurcation of model (0.2) at the interior fixed point

$$p_1\left( \frac{\gamma }{\alpha - \gamma }, \frac{r}{\alpha - \gamma }e^{\frac{\alpha - 2\gamma }{\alpha - \gamma }} \right) ,$$

as the parameters vary in a small neighborhood. Let $(r^*, \alpha , \gamma )$ be arbitrary parameters, and take $r^*$ as a new dependent bifurcation parameter. The system then becomes:

2.5 $$\begin{aligned} x_{n+1}&= x_n + (r + r^*)x_n e^{1 - x_n} - \frac{\alpha x_n y_n}{1 + x_n}, \nonumber \\ y_{n+1}&= y_n + \frac{\alpha x_n y_n}{1 + x_n} - \gamma y_n. \end{aligned}$$

Define $u_n = x_n - x^*$ and $v_n = y_n - y^*$ to shift the fixed point $p_1$ to the origin. After computation, we obtain the following expressions:

$$\begin{aligned} u_{n+1}= & u_{n}+\left( u_{n}+x^{*}\right) e^{1-u_{n}-x^{*}}\left( r+r^{*}\right) -\frac{\alpha \left( u_{n}+x^{*}\right) \left( v_{n}+y^{*}\right) }{1+u_{n}+x^{*}}, \\ v_{n+1}= & v_{n}+\frac{\alpha \left( u_{n}+x^{*}\right) \left( v_{n}+y^{*}\right) }{1+u_{n}+x^{*}}-\gamma \left( v_{n}+y^{*}\right) . \end{aligned}$$

2.6 $$\begin{aligned} u_{n+1}&= \widetilde{B_{11}}u_n + \widetilde{B_{12}}v_n + \widetilde{B_{13}}u_n^2 + \widetilde{B_{14}}u_n v_n + \widetilde{B_{15}}u_n^3 + \widetilde{B_{16}}u_n^2 v_n + \widetilde{B_{17}}u_n r^* + \widetilde{B_{18}}u_n^2 r^* + O\left( |u_n|, |v_n|, |r^*| \right) ^4,\nonumber \\ v_{n+1}&= \widetilde{B_{21}}u_n + \widetilde{B_{22}}v_n + \widetilde{B_{23}}u_n^2 + \widetilde{B_{24}}u_n v_n + \widetilde{B_{25}}u_n^3 + \widetilde{B_{26}}u_n^2 v_n + O\left( |u_n|, |v_n| \right) ^4. \end{aligned}$$

The coefficients are given by:

$$\begin{aligned} \widetilde{B_{11}}&= \frac{-r^*(x^* - 1)(x^* + 1)^2 e^{1 - x^*} - \alpha y^*}{(1 + x^*)^2} + 2, \quad&\widetilde{B_{12}}&= \frac{-\alpha x^*}{1 + x^*}, \\ \widetilde{B_{13}}&= \frac{(x^* + 1)^3 (x^* - 2) r^* e^{1 - x^*} + 2\alpha y^*}{2(x^* + 1)^3}, \quad&\widetilde{B_{14}}&= \frac{-\alpha }{1 + x^*}, \\ \widetilde{B_{15}}&= \frac{-(x^* + 1)^4 (x^* - 3) r^* e^{1 - x^*} - 6\alpha y^*}{6(1 + x^*)^4}, \quad&\widetilde{B_{16}}&= \frac{\alpha }{(x^* + 1)^3}, \\ \widetilde{B_{17}}&= (1 - x^*) e^{1 - x^*}, \quad&\widetilde{B_{18}}&= \frac{(x^* - 2)e^{1 - x^*}}{2}, \\ \widetilde{B_{21}}&= \frac{\alpha y^*}{(x^* + 1)^2}, \quad&\widetilde{B_{22}}&= \frac{\alpha x^* + x^* + 1}{x^* + 1}, \\ \widetilde{B_{23}}&= \frac{-\alpha y^*}{(x^* + 1)^3}, \quad&\widetilde{B_{24}}&= \frac{\alpha }{(x^* + 1)^3}, \\ \widetilde{B_{25}}&= \frac{\alpha y^*}{(x^* + 1)^4}, \quad&\widetilde{B_{26}}&= \frac{-\alpha }{(x^* + 1)^3}. \end{aligned}$$

Define the invertible matrix:

$$M = \begin{pmatrix} \widetilde{B_{12}} & \widetilde{B_{12}}\\ -1 - \widetilde{B_{11}} & \rho _2 - \widetilde{B_{11}} \end{pmatrix},$$

and apply the coordinate transformation:

$$\begin{pmatrix} u_n \\ v_n \end{pmatrix} = M \begin{pmatrix} X_n \\ Y_n \end{pmatrix}.$$

Then the system becomes

2.7 $$\begin{aligned} \begin{pmatrix} X_{n+1} \\ Y_{n+1} \end{pmatrix} = \begin{pmatrix} -1 & 0 \\ 0 & \rho _2 \end{pmatrix} \begin{pmatrix} X_n \\ Y_n \end{pmatrix} + \begin{pmatrix} \widetilde{\Psi }(u_n, v_n, r^*) \\ \widetilde{\Phi }(u_n, v_n, r^*) \end{pmatrix}. \end{aligned}$$

Here, $\widetilde{\Psi }$ and $\widetilde{\Phi }$ are:

$$\begin{aligned} \widetilde{\Psi }&= \frac{\widetilde{B_{13}}(\rho _2 - \widetilde{B_{11}}) - \widetilde{B_{12}} \widetilde{B_{23}}}{\widetilde{B_{12}}(1 + \rho _2)} u_n^2 + \frac{\widetilde{B_{14}}(\rho _2 - \widetilde{B_{11}}) - \widetilde{B_{12}} \widetilde{B_{24}}}{\widetilde{B_{12}}(1 + \rho _2)} u_n v_n \\&\quad + \frac{\widetilde{B_{17}}(\rho _2 - \widetilde{B_{11}})}{\widetilde{B_{12}}(1 + \rho _2)} u_n r^* + \frac{\widetilde{B_{18}}(\rho _2 - \widetilde{B_{11}})}{\widetilde{B_{12}}(1 + \rho _2)} u_n^2 r^* + \frac{\widetilde{B_{15}}(\rho _2 - \widetilde{B_{11}}) - \widetilde{B_{25}} \widetilde{B_{12}}}{\widetilde{B_{12}}(1 + \rho _2)} u_n^3 \\&\quad + \frac{\widetilde{B_{16}}(\rho _2 - \widetilde{B_{11}}) - \widetilde{B_{26}} \widetilde{B_{12}}}{\widetilde{B_{12}}(1 + \rho _2)} u_n^2 v_n + O(|u_n|, |r^*|)^4, \\ \widetilde{\Phi }&= \frac{\widetilde{B_{13}}(1 + \widetilde{B_{11}}) + \widetilde{B_{12}} \widetilde{B_{23}}}{\widetilde{B_{12}}(1 + \rho _2)} u_n^2 + \frac{\widetilde{B_{14}}(1 + \widetilde{B_{11}}) + \widetilde{B_{12}} \widetilde{B_{24}}}{\widetilde{B_{12}}(1 + \rho _2)} u_n v_n \\&\quad + \frac{\widetilde{B_{17}}(1 + \widetilde{B_{11}})}{\widetilde{B_{12}}(1 + \rho _2)} u_n r^* + \frac{\widetilde{B_{18}}(1 + \widetilde{B_{11}})}{\widetilde{B_{12}}(1 + \rho _2)} u_n^2 r^* + \frac{\widetilde{B_{15}}(1 + \widetilde{B_{11}}) + \widetilde{B_{25}} \widetilde{B_{12}}}{\widetilde{B_{12}}(1 + \rho _2)} u_n^3 \\&\quad + \frac{\widetilde{B_{16}}(1 + \widetilde{B_{11}}) - \widetilde{B_{26}} \widetilde{B_{12}}}{\widetilde{B_{12}}(1 + \rho _2)} u_n^2 v_n + O(|u_n|, |r^*|)^4. \end{aligned}$$

The nonlinear terms are further transformed using:

$$\begin{aligned} u_n^2&= \widetilde{B_{12}}^2 (X_n^2 + 2X_n Y_n + Y_n^2), \\ u_n v_n&= -\widetilde{B_{12}}(1 + \widetilde{B_{11}})X_n^2 + \widetilde{B_{12}}(\rho _2 - 2\widetilde{B_{11}} - 1)X_n Y_n + \widetilde{B_{12}}(\rho _2 - \widetilde{B_{11}})Y_n^2, \\ u_n r^*&= \widetilde{B_{12}}(X_n + Y_n)r^*, \\ u_n^2 r^*&= \widetilde{B_{12}}^2 (X_n^2 + 2X_n Y_n + Y_n^2) r^*. \end{aligned}$$

By applying the center manifold theorem^21^, the center manifold $T^c(0,0)$ of(2.7) around $r^*$ is given by:

$$T^c(0,0) = \left\{ (X_n, Y_n, r^*) \mid Y_n = Z(X_n, r^*) = k_0 r^* + k_1 X_n^2 + k_2 X_n r^* + k_3 (r^*)^2 + O(|u_n|, |r^*|)^3 \right\} ,$$

with coefficients:

$$\begin{aligned} k_0&= 0, \quad k_3 = 0, \\ k_1&= \frac{(1 + \widetilde{B_{11}})\left[ \widetilde{B_{14}}(1 + \widetilde{B_{11}}) - \widetilde{B_{12}}(\widetilde{B_{13}} - \widetilde{B_{24}}) \right] - \widetilde{B_{23}}\widetilde{B_{12}}^2}{\rho _2^2 - 1}, \\ k_2&= \frac{[\widetilde{B_{18}}(1 + \widetilde{B_{11}}) - \widetilde{B_{17}}\widetilde{B_{12}}](1 + \widetilde{B_{11}})}{\widetilde{B_{12}}(1 + \rho _2)^2}. \end{aligned}$$

Substituting into (2.7), the reduced dynamics on the center manifold becomes:

2.8 $$\begin{aligned} L(X_n) = -X_n + n_1 X_n^2 + n_2 X_n r^* + n_3 X_n^2 r^* + n_4 X_n (r^*)^2 + n_5 X_n^3 + O(|X_n|, |r^*|)^4, \end{aligned}$$

where the coefficients $n_1, \ldots , n_5$ are derived via substitution.

$$n_{1}=\frac{1}{1+\rho _{2}}\left[ \widetilde{B_{14}}\widetilde{B_{11}}^{2}+ \widetilde{B_{11}}\left( \widetilde{B_{12}}\left( \widetilde{B_{24}}- \widetilde{B_{13}}\right) -\left( \rho _{2}-1\right) \widetilde{B_{14}} \right) +\widetilde{B_{12}}\left( \rho _{2}\widetilde{B_{13}}+\widetilde{ B_{24}}\right) -\widetilde{B_{23}}\widetilde{B_{12}}^{2}-\rho _{2}\widetilde{ B_{14}}\right] ,$$

$$n_{2}=\frac{1}{\left( 1+\rho _{2}\right) \widetilde{B_{12}}}\left[ \left( \widetilde{B_{12}}\widetilde{B_{17}}-\left( 1+\widetilde{B_{11}}\right) \widetilde{B_{18}}\right) \left( \rho _{2}-\widetilde{B_{11}}\right) \right] ,$$

$$n_{3}=\frac{1}{\left( \rho _{2}-1\right) \left( 1+\rho _{2}\right) ^{3} \widetilde{B_{12}}}[\left( 1+\widetilde{B_{11}}\right) \left( 2\widetilde{ B_{12}}^{3}\widetilde{B_{23}}\widetilde{B_{15}}\left( \rho _{2}-1\right) +((( \widetilde{B_{24}}-2\widetilde{B_{13}}\right) \widetilde{B_{15}}-\widetilde{ B_{17}}(\widetilde{B_{13}}-\widetilde{B_{23}})\rho _{2}^{2}$$

$$+(((-2\widetilde{B_{24}}+2\widetilde{B_{13}})\widetilde{B_{15}}+\widetilde{ B_{17}}(\widetilde{B_{13}}-\widetilde{B_{23}})\widetilde{B_{11}}-2\left( \widetilde{B_{24}}-\widetilde{B_{13}}\right) \widetilde{B_{15}}-\widetilde{ B_{17}}(\widetilde{B_{13}}-\widetilde{B_{23}}))\rho _{2}+(2\widetilde{B_{15}}$$

$$\left( \widetilde{B_{24}}-\widetilde{B_{13}}\right) +\widetilde{B_{17}}( \widetilde{B_{13}}-\widetilde{B_{23}}))\widetilde{B_{11}}+\widetilde{B_{24}} \widetilde{B_{15}})\widetilde{B_{12}}^{2}-(\rho _{2}-\widetilde{B_{11}})( \widetilde{B_{14}}\widetilde{B_{15}}+\widetilde{B_{18}}(\widetilde{B_{13}}- \widetilde{B_{23}}))\rho _{2}^{2}],$$

$$n_{4}=\frac{1}{\left( 1+\rho _{2}\right) ^{3}\widetilde{B_{12}}}\left[ \widetilde{B_{15}}\left( 1+\widetilde{B_{11}}\right) \left( \widetilde{B_{18} }\rho _{2}-\widetilde{B_{18}}\widetilde{B_{11}}+\widetilde{B_{12}}\widetilde{ B_{17}}\right) (\widetilde{B_{11}}-\rho _{2})\right] ,$$

$$n_{5}=\frac{1}{\left( \rho _{2}-1\right) \left( 1+\rho _{2}\right) ^{2}}[(2 \widetilde{B_{23}}\widetilde{B_{11}}(\widetilde{B_{13}}-\widetilde{B_{23}} )-\rho _{2}^{2}\widetilde{B_{25}}+2\widetilde{B_{23}}\widetilde{B_{13}}-2 \widetilde{B_{23}}^{2}+\widetilde{B_{25}})\widetilde{B_{12}}^{3}$$

$$+((-2\widetilde{B_{23}}\widetilde{B_{14}}+2(-\widetilde{B_{13}}-\widetilde{ B_{24}})(\widetilde{B_{13}}-\widetilde{B_{23}}))\widetilde{B_{11}}^{2}+(( \widetilde{B_{26}}-\widetilde{B_{15}})\rho _{2}^{2}-2(\widetilde{B_{13}}- \widetilde{B_{23}})$$

$$\left( \widetilde{B_{13}}-\frac{1}{2}\widetilde{B_{24}}\right) \rho _{2}-4 \widetilde{B_{14}}\widetilde{B_{23}}+\widetilde{B_{23}}\left( 3\widetilde{ B_{24}}-2\widetilde{B_{13}}\right) +2\widetilde{B_{13}}^{2}-3\widetilde{ B_{13}}\widetilde{B_{24}}-\widetilde{B_{26}}+\widetilde{B_{15}})\widetilde{ B_{11}}$$

$$+\rho _{2}^{3}\widetilde{B_{15}}+\rho _{2}^{2}\widetilde{B_{26}}+\left( \left( 2\widetilde{B_{13}}-\widetilde{B_{24}}\right) \widetilde{B_{23}}-2 \widetilde{B_{13}}^{2}+\widetilde{B_{13}}\widetilde{B_{24}}-\widetilde{B_{15} }\right) \rho _{2}-\widetilde{B_{24}}\widetilde{B_{13}}-2\widetilde{B_{23}} \widetilde{B_{14}}$$

$$+\widetilde{B_{23}}\widetilde{B_{24}}-\widetilde{B_{26}})\widetilde{B_{12}} ^{2}.$$

To be the map $L\left( X_{n}\right)$ a period-doubling bifurcation, we require that the following conditions are non-zero:

$$\begin{aligned} \varpi _{1}= & \left( \frac{\partial ^{2}L}{\partial X_{n}\partial r^{*}} +\frac{1}{2}\frac{\partial L}{\partial r^{*}}\frac{\partial ^{2}L}{ \partial X_{n}^{2}}\right) |_{\left( 0,0\right) }, \\ \varpi _{2}= & \left( \frac{1}{6}\frac{\partial ^{3}L}{\partial X_{n}^{3}} +\left( \frac{1}{2}\frac{\partial ^{2}L}{\partial X_{n}^{2}}\right) ^{2}\right) |_{_{\left( 0,0\right) }}, \end{aligned}$$

by simple computation

$$\varpi _{1}=n_{2}\ne 0,$$

and

$$\varpi _{2}=n_{5}+n_{1}^{2}\ne 0.$$

#### Proposition 2.2

Assuming $\varpi _{2}\ne 0$, the map (0.2) undergoes a period-doubling bifurcation about the positive equilibrium $p_{1}$ when $r^{*}$varies in a small neighborhood of $O\left( 0,0\right)$, in addition to, if $\varpi _{2}>0$ (resp. $\varpi _{2}<0$), so the period-2 points that bifurcate from $p_{1}$ are stable (resp. unstable).

## Numerical study

 We use numerical analysis to produce phase portraits, Lyapunov exponent and bifurcation diagrams of model (0.2), which validate the earlier theoretical analysis and uncover novel complicated dynamical features (see figures (), ()). To determine points of interest and get bifurcation curves of the system (0.2) for various parameter values, we use Matlab to execute numerical continuation of equilibria. In the next three scenarios, we take the bifurcation parameters into consideration.

**Case 1**: With $\alpha =1$ and $\gamma =.5$ as the parameters and the initial value $\left( x_{0},y_{0}\right) =\left( 0.33,0.64\right)$, the two-dimensional predator–prey model (0.2) has a stable equilibrium point $p_{0}(0,0)$, which indicates that there are no predators or prey in the area if $r<5.24415$. In the case when $r=5.24415$, it becomes unstable. The description of equilibrium $p_{0}(0,0)$ in Sect. “Introduction” is shown to be accurate in Fig. 3.

**Fig. 1:**
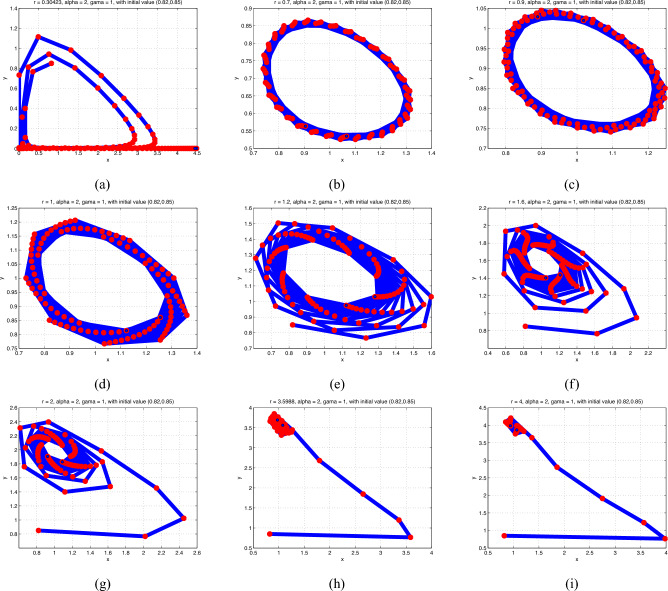
Phase portraits of model (2.1) for various values of *r* from.30423 to 4.

**Case 2**: By fixing $\alpha =2$ and $\gamma =1$ and altering r in the range $.3<r\leqslant 4$, we find that a Neimark-Sacker bifurcation occurs for $r=.30423$. Model (0.2) has a unique positive stable equilibrium point at $r=3.598768$. For example, we determine that (1, 3.598768) is the unique equilibrium point for $\left( \alpha ,\gamma ,r\right) =\left( 2,1,3.598768\right)$. Fig.1 displays the appropriate phase portraits for a range of *r* values. From the numerical results shown in Fig. 1, it can be showed that the stable equilibrium $p_{1}$ loses its stability, leading to the emergence of a persistent and positive periodic oscillation that permits communities of predators and prey coexisting over time.

**Case 3**: We fixed $\alpha =.5$ and $\gamma =.3$ and ran numerical simulations by changing the value of *r* in the range $3< r<6$. The findings are shown in Fig. 4. For $r<3.93311$, we find that $p_{1}$ stays stable and that the prey and predator densities are stabled at the stable equilibrium point $p_{1}$. But when $r=3.93311$, a period-doubling bifurcation causes $p_{1}$ to lose stability, leading to stable periodic oscillations of period 2. Stable periodic orbits of periods 4, 8, and so forth result from additional period-doubling bifurcations that occur in the system as *r* rises above the bifurcation point. The system exhibits chaotic behavior when a chaotic set finally appears for a few larger values of *r*. The bifurcation diagram and maximal Lyapunov exponent of the system (0.2) may be obtained to further validate that statement.

**Fig. 2:**
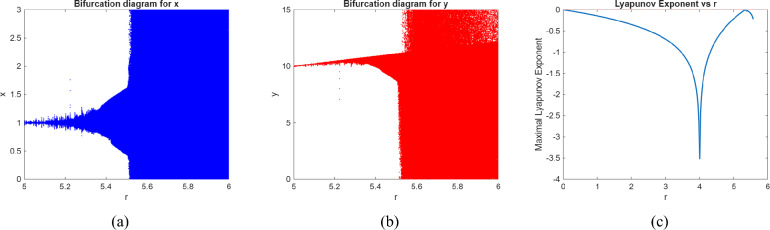
Bifurcation diagram and Maximum Lyapunov exponent of model (0.2) for $\alpha =1$ and $\gamma =.5$.

**Fig. 3:**
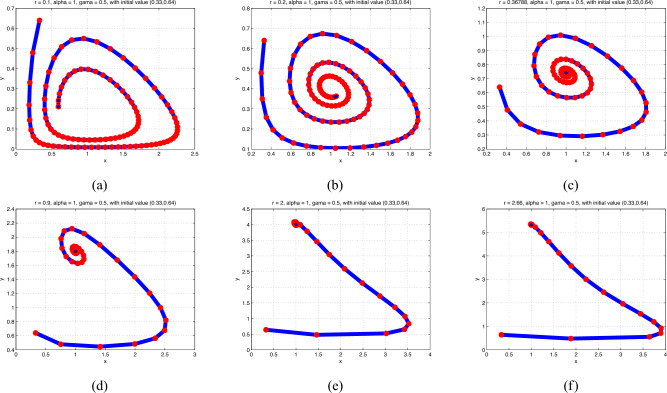
Phase portraits of model (0.2) for various values of *r* from.1 to 2.66.

**Fig. 4:**
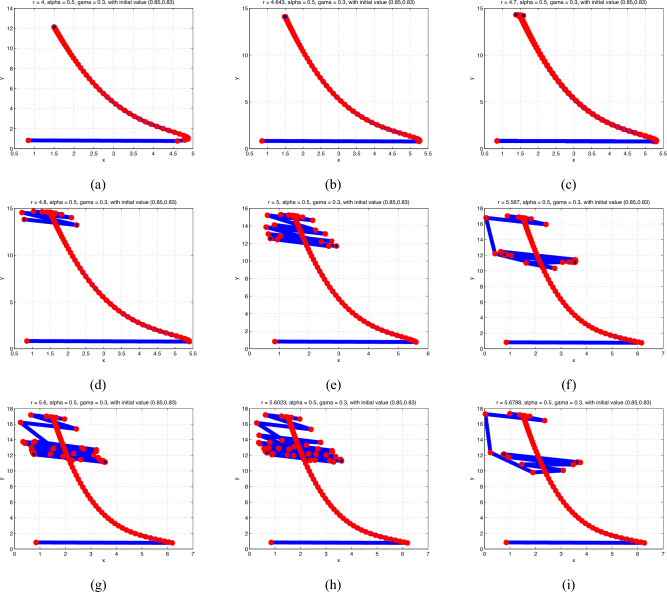
Phase portraits of model (2.5) for various values of *r* from 4 to 5.6789.

**Fig. 5:**
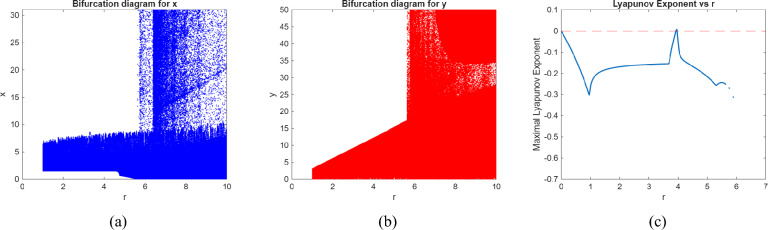
Bifurcation diagram and Maximum Lyapunov exponent of model (2.5) for $\alpha =.5$ and $\gamma =.3$.

**Fig. 6:**
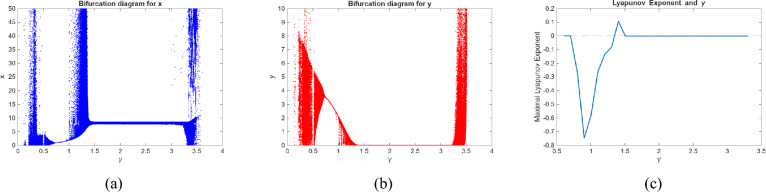
Bifurcation diagram and Maximum Lyapunov exponent of model (0.2) for $r =2.64$ and $\alpha =1.55$.

## Conclusion and critical discussion

 Our study of the dynamics of a two-dimensional predator-prey model with a Holling type II functional response has, in summary, revealed interesting chaotic behavior and bifurcation phenomena such as Neimark-Sacker and period-doubling bifurcations. A thorough illustration of numerous bifurcations and equilibrium point stability regions in a single graphic offers a concise summary of the intricate dynamics of the model. The bifurcation and Lyapunov exponent analyses reveal transitions from stable equilibria to periodic and chaotic regimes as the intrinsic growth rate *r* increases. Natural death was included for predators in order to compare the biological comparison of models (0.1) and (0.2). Changes in *r*, $\alpha$ and $\gamma$ will result in Neimark–Sacker bifurcations and period-doubling to (0.2).

 As is characteristic of nonlinear ecological systems, these transitions show that small change to parameters can have significant effects on population dynamics. Even though the model is helpful for investigating basic dynamical patterns, it has a number of limitations that need to be carefully considered.

Simplified Interaction Structure: the interaction coefficient $\frac{\alpha x_{n} y_{n}}{1 + x_{n}}$ simplifies actual predator feeding behaviors by assuming a saturating (Holling type II) functional response. Predator efficiency may actually be influenced by handling time or prey availability, and constant Parameters, whereas ecological parameters vary with environmental conditions, resource availability, and seasonality. This restricts its ability to predict realistic, time-varying ecosystem dynamics.

 The findings point to numerous significant consequences for a biological explanation, including population uncertainty and chaotic regimes. Long-term forecasts are unreliable due to the sensitive dependency on initial conditions indicated by positive Lyapunov exponents, which suggest that little changes in the environment or population composition might result in significant population changes. The regions of parameter space with negative Lyapunov exponents that correspond to stable coexistence indicate circumstances in which both populations may exist in a sustainable manner. Understanding how growth rate and interaction strength affect stability can be useful for managing ecosystems, such as preventing over exploitation or preserving the balance between predators and prey.

 In summary, while the model effectively captures essential nonlinear predator-prey interactions and provides insight into routes to chaos, its simplifications limit direct ecological applicability. Future extensions could incorporate environmental variability, stochastic effects, and multi-species interactions to enhance realism and deepen ecological interpretation.

## Data Availability

All data generated or analysed during this study are included in this published article.
